# Impact of inability to turn in bed assessed by a wearable three-axis accelerometer on patients with Parkinson's disease

**DOI:** 10.1371/journal.pone.0187616

**Published:** 2017-11-09

**Authors:** Kenji Uchino, Makoto Shiraishi, Keita Tanaka, Masashi Akamatsu, Yasuhiro Hasegawa

**Affiliations:** Department of Neurology, St. Marianna University School of Medicine, Kawasaki, Kanagawa, Japan; Charite Medical University Berlin, GERMANY

## Abstract

**Background:**

Difficulty turning over in bed is a common night-time symptom in Parkinson’s disease (PD). We aimed to quantitatively evaluate overnight turnover movements using a three-axis accelerometer and to investigate whether inability to turn in bed is related to daytime sleepiness, sleep quality, and depressive mood in PD patients.

**Methods:**

We examined 64 patients with PD (mean age, 73.3±8.21 years; modified Hoehn-Yahr [mH-Y] stage, 3.0±1.0; disease duration, 7.2±6.3 years; unified Parkinson's disease rating scale [UPDRS], 36.9±18.3). Overnight monitoring of turnover movements using a wearable three-axis accelerometer was performed in all patients. Nocturnal kinetic parameters including total time recumbent, total time supine, number of turnover movements, and mean interval between turnover movements were obtained. Daytime immobility was assessed using the Barthel index (B-I), UPDRS, and mH-Y stage. Patients were also assessed with the Epworth Sleepiness Scale (ESS), Parkinson’s Disease Sleep Scale-2 (PDSS-2), and Beck Depression Inventory (BDI).

**Results:**

Number of turnover movements in bed correlated negatively with disease duration (r = -0.305; p<0.05), L-dopa-equivalent dose (r = -0.281; p<0.05), mH-Y staging (r = -0.336; p<0.01), total score of UPDRS (r = -0.386; p<0.01) and positively with B-I score (r = 0.365; p<0.01). Number of turnover movements in bed was generally inconsistent with awareness of turnover movement impairment as evaluated by PDSS-2 Item 9 scores, but patients who were never aware of impaired turnover movements showed ≥5 turnover movements overnight. Multivariate logistic regression analyses revealed no correlations between number of nocturnal turnover movements in bed and BDI, ESS, or PDSS-2. Use of anti-psychotic drugs was associated with ESS (p = 0.045). UPDRS was associated with PDSS-2 (p = 0.016).

**Conclusion:**

Decreased number of turnover movements may not be a direct determinant of daytime sleepiness, sleep disorders, or depressive mood in PD patients. Use of anti-psychotic drugs and higher UPDRS score are factors significantly associated with daytime sleepiness and uncomfortable sleep, respectively.

## Introduction

Difficulty turning over in bed is one of the motor symptoms of Parkinson’s disease (PD), with a prevalence of 45–82% for this subjective complaint [1–4]. Nocturnal movement and sleep disorders such as rapid eye movement sleep behavior disorder in PD patients have been extensively studied using polysomnography [5–7]. Whether this nocturnal motor deficit affects the quality of life of PD patients thus remains unclear. In recent years, the accuracy of three-axis accelerometers has improved together with advances in miniaturization, and wearable accelerometers are now available to analyze kinetic data of overnight movements in PD patients [8–11]. This sensor-based kinetic analysis may shed light on the real clinical impact of difficulty turning over in bed among PD patients. This study set out to objectively assess nocturnal turnover movements in PD patients with overnight monitoring using a wearable three-axis accelerometer to test the hypothesis that inability to turn in bed would negatively impact on daytime sleepiness, sleep quality, and depressive mood in PD patients.

## Patients and methods

### Study population

We examined PD patients who met the following inclusion criteria: 1) fulfilment of the diagnostic criteria described by the UK Parkinson Disease Society brain bank [12]; 2) age ≥18 years; 3) provision of written informed consent to participate in this study; 4) treatment with the same antiparkinsonian drug during the 2 weeks before entry; and 5) ability to comply with the study protocol. We excluded patients with: 1) serious concurrent disease such as severe inflammatory disease (white blood cell count > 1.0 ×10^4^ mm^3^; C-reactive protein > 3.0 mg/dl); 2) hallucination or delusion; or 3) dementia (Mini-Mental State Examination [MMSE] <16). No restrictions were placed on disease duration. This study was conducted in a single hospital, and the study protocol was approved by the St. Marianna University Bioethics Committee (approval no. 2487). All participants provided written informed consent.

### Overnight monitoring of turnover movements in bed

**Fig 1:**
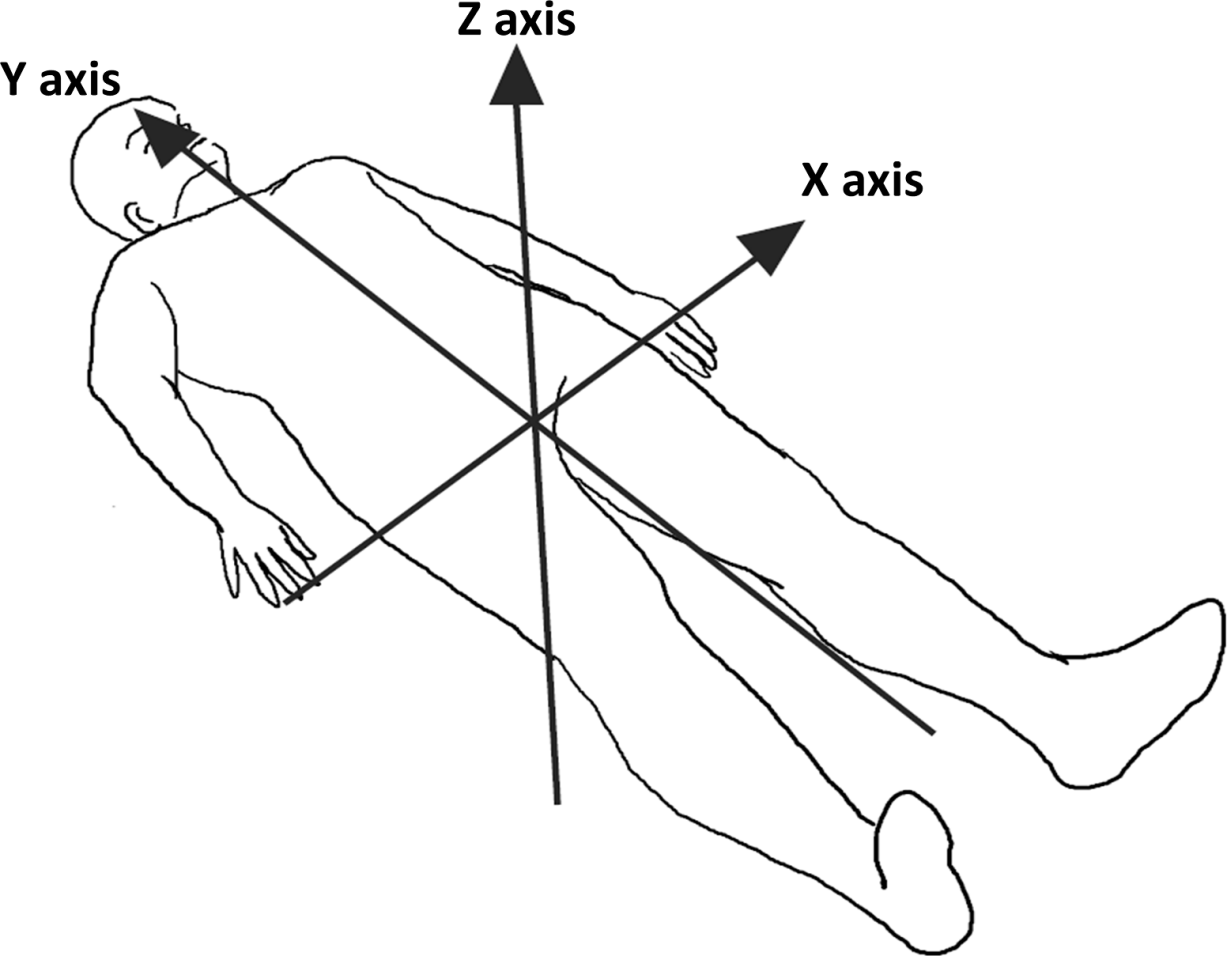
Three-axis accelerometer sensor axes in the recumbent position. X axis: from right to left; Y axis: from caudal to rostral; Z axis: from back to front.

We used a published method for assessing turnover movements in bed with the three-axis accelerometer [8]. To state the method in brief, a wearable motion recorder (75 mm × 50 mm × 20 mm, 120 g) equipped with three-axis acceleration sensors (Mimamori-Gait system, MG-M1110-HW; LSI Medience, Tokyo, Japan) was fixed on the center of the abdomen at the navel using a special belt (Fig 1). Three-axis acceleration data were collected at 100 Hz and serially stored in the micro Secure Digital memory card of the recorder through A/D conversion. All patients were monitored for 10 h from 21:00 to 07:00 the next day, and data were analyzed offline using commercially available software (MG-M1100-PC; LSI Medience). The nocturnal kinetic parameters used in this study were: 1) total time recumbent; 2) total time supine; 3) number of turnover movements; and 4) mean interval between turnover movements.

**Fig 2:**
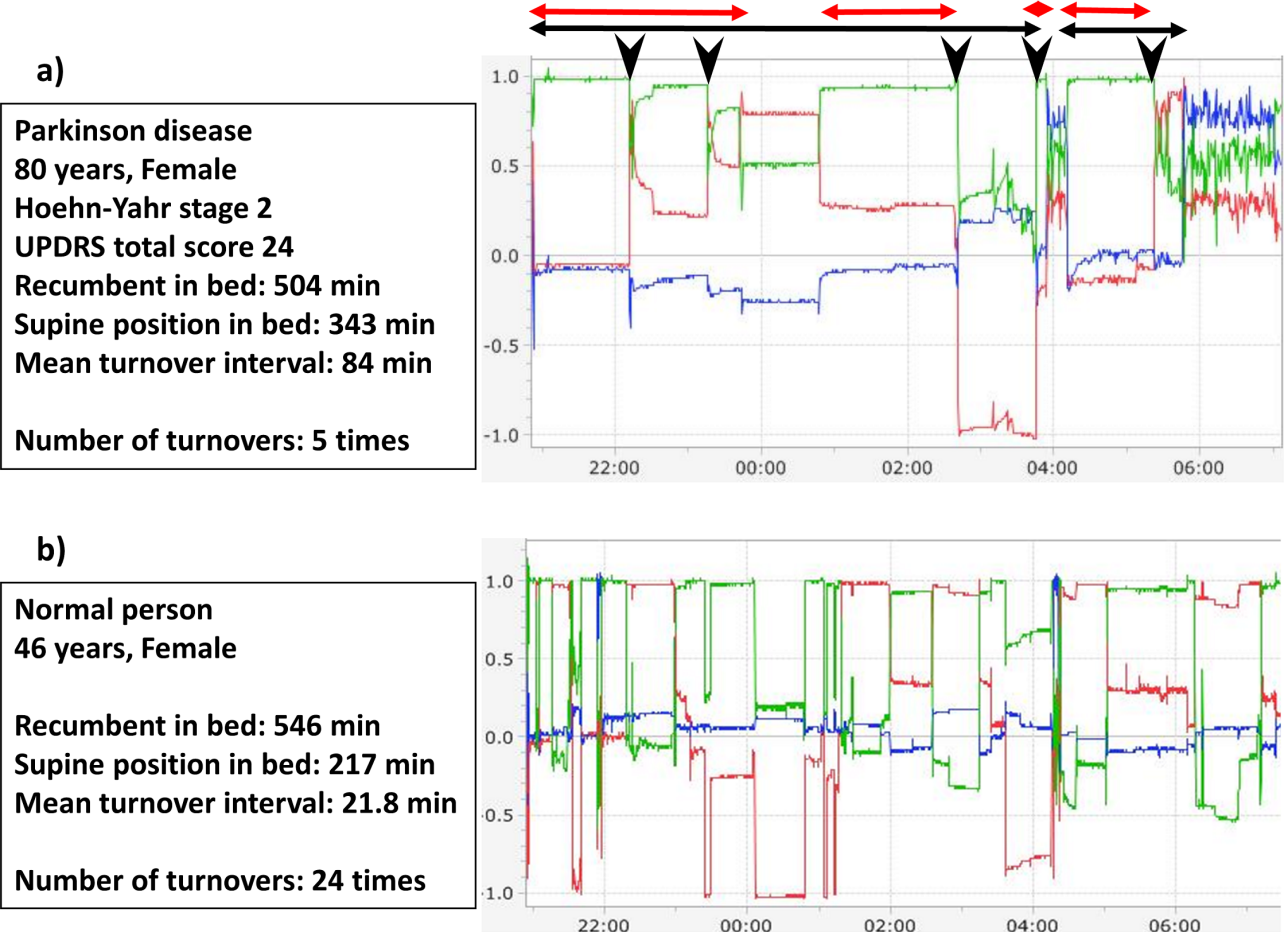
Number of turnover movements in bed in PD patients and healthy subjects. Representative overnight recordings from 21:00 to 07:00 the following day are shown. a) Chart from a PD patient. Arrowhead indicates turnover movement, black lines indicate time recumbent, and red lines indicate time supine. b) Chart from a healthy subject. Variations in acceleration along the X, Y, and Z axes are represented by red, blue, and green lines, respectively.

Typical overnight monitoring charts are shown in Fig 2. Variations in acceleration along the X, Y, and Z axes are represented by red, blue, and green lines, respectively. As a turnover movement in bed is a movement around the Y axis, acceleration in the Y axis direction remains essentially zero (blue lines in Fig 2A and Fig 2B). Turnover movements in bed were defined as movements simultaneously registering acceleration along the three axes as follows: ≥0.58 G for the X axis; ≤0.32 G for the Y axis; and ≥0.20 G for the Z axis. These cut-off values were derived based on a 95% confidence interval to detect patient rotations ≥30° around the center of the anteroposterior axis as turnover movements in bed [8]. Accordingly, turnover movements with angles of rotation <30° were not regarded as turnover movements in bed. Turnover movements in bed can thus be counted based on the typical turnover movements followed by a stable period (Fig 2).

Once a turnover movement occurs, body position remains stable for a variable duration from a few minutes to hours. Evaluating not only the number of turnover movements but also the pattern of appearance of turnover movements is important. Some PD patients showed several clusters of turnover movement with short duration and staying in supine sleep for a long time. We thus introduced the mean interval between turnover movements in this study. The position of the patient (i.e., recumbent, sitting, or standing) based on the waveform of the Y axis is also easy to determine. Total measurement period was 600 min, and the amount of time the patient stayed in an upright position (acceleration on the Y axis ≈ 1.0), which was assumed to represent the patient being awake and out of bed, was subtracted from this total measurement period to obtain the duration spent in bed in a recumbent position, i.e., total time recumbent. We also obtained the total time supine, because several studies have shown that the supine sleep of PD patients is associated with increased obstructive sleep apnea, suggesting that for individuals with PD, alleviating the difficulties of turning over in bed might reduce time spent in the supine sleep position and improve sleep-disordered breathing [13].

The reliability of measurement results was verified by overnight monitoring for 11 PD patients (5 men; mean age, 76.1 years; median Hoehn and Yahr (H-Y) scale measurement, 4 [2–5]) twice, at an interval of 2 days. For the number of turnover movements, these measurements yielded an intraclass correlation coefficient of 0.737 (p = 0.019).

### Clinical data collection

Clinical characteristics of each patient in the study were assessed by a certified neurologist. The clinical background of each patient (age, disease duration, initial presentation, and oral medication) was checked. The dose of antiparkinsonian drugs (levodopa [L-dopa], dopamine agonists, and catechol-O-methyltransferase [COMT] inhibitors) was converted into an L-dopa-equivalent dose (LED), as necessary [14]. We also established whether patients were taking drugs that could be expected to affect sleep or depression such as sleeping medication, anti-depressants, anti-psychotic drugs, or amantadine. Severity of PD was assessed using a modified H-Y (mH-Y) staging scale [15] and the UPDRS [16]. Activities of daily living were assessed using the Barthel index (B-I) [17]. UPDRS assessments included the total score and sub-scores for Parts 2 and 3.

Sleepiness, awareness of sleep disturbance, and depressive features were assessed with the Epworth Sleepiness Scale (ESS) [18], Parkinson’s Disease Sleep Scale-2 (PDSS-2) [19, 20], and the Beck Depression Inventory (BDI) [21], respectively. Subjective awareness of difficulty turning in bed in each patient was classified into five categories from Score 0 to Score 4 using a question in PDSS-2 item 9 (“Did you feel uncomfortable at night because you were unable to turn in bed or move due to immobility?”). Possible responses to this question were scored as: 0, “never”; 1, “occasionally (1 night/week)”; 2, “sometimes (2–3 nights/week)”; 3, “often (4–5 nights/week)”; or 4, “very often (6–7 nights/week)”.

### Statistical analyses

Characteristics of patients are given as the mean and standard deviation (SD), unless otherwise indicated. Unpaired Student’s *t* test was used to compare continuous variables, and χ^2^ tests were used for nominal parameters. The Mann-Whitney U test and Kruskal-Wallis test were used for data that did not show normal distributions. Relationships between clinical scales and nocturnal kinetic parameters were investigated using Spearman’s rank correlation analysis. Overnight frequency of turnover movement in patients with PD was divided into quartiles and logistic regression models adjusted for potential confounders including age, disease duration, and mH-Y scale score were used to estimate odds ratios with 95% confidence intervals for associations with the first quartile (i.e., lowest frequency group). For logistic regression analysis, ESS scores ≥10 were taken to indicate excessive daytime sleepiness [18], PDSS-2 scores ≥15 were taken to indicate sleep disturbance [22], and BDI scores >20 were taken to indicate moderate to severe depressive features [21]. Two-sided *p-*values <0.05 were considered statistically significant. All statistical analyses were performed using SPSS Statistics for Windows version 21 software (IBM, Armonk, NY).

## Results

We examined 65 patients with PD from April 2013 to July 2016. Overnight monitoring of turnover movements was performed in all patients. We excluded 1 patient for whom overnight recordings were discontinued because of hallucinations. A total of 64 patients (35 men; mean age, 73.3±8.21 years) were analyzed. Patient demographic characteristics are shown in Table 1. Daytime assessment results were as follows: B-I, 76.6±27.4; UPDRS, 36.9±18.3 (range, 4–88); BDI, 15.5±12.4 (range, 0–43); ESS, 7.03±4.55 (range, 0–19); and PDSS-2, 19.3±9.96 (range, 1–49). Mean LED for patients was 540±317 mg (range, 0–1265 mg). Thirty-five patients (54.7%) were treated with dopamine agonist, with pramipexole as the most widely used medication (17 patients, 26.6%). Eleven patients were taking anti-psychotic drugs to prevent hallucinations, and eight patients were taking antidepressants.

**Table 1:** Patient characteristics.

	Patients with PD n = 64
Age, mean (SD)	73.3 (8.21)
Male sex, n (%)	35 (54.7)
Height, m (SD)	159 (9.77)
Weight, kg (SD)	54.2 (12.2)
BMI, kg/m^2^ (SD)	21.2 (3.65)
Age at onset, years (SD)	66.1 (10.1)
Disease duration, years (SD)	7.22 (6.28)
Modified Hoehn-Yahr staging (SD)	3.0 (1.0)
Barthel index (SD)	76.6 (27.4)
Unified Parkinson’s Disease Rating Scale	
Total score	36.9 (18.3)
Part 1	2.61 (2.03)
Part 2	10.9 (6.76)
Part 3	19.9 (11.4)
Part 4	2.39 (2.99)
Beck Depression Inventory	15.5 (12.4)
Epworth Sleepiness Scale	7.03 (4.55)
Parkinson’s Disease Sleep Scale-2	19.3 (10.0)
LED, mg (SD)	540 (317)
Dopamine agonist, n (%)	35 (54.7)
Pramipexole, n (%)	17 (26.6)
Rotigotine, n (%)	8 (12.5)
Ropinirole, n (%)	11 (17.2)
Ergot, n (%)	1 (1.56)
Amantadine, n (%)	9 (14.1)
Sleeping drug, n (%)	15 (23.4)
Antipsychotic drug, n (%)	11 (17.2)
Antidepressant, n (%)	8 (12.5)

LED, L-dopa-equivalent dose; SD, standard deviation.

### Turnover movements and severity of PD

**Fig 3:**
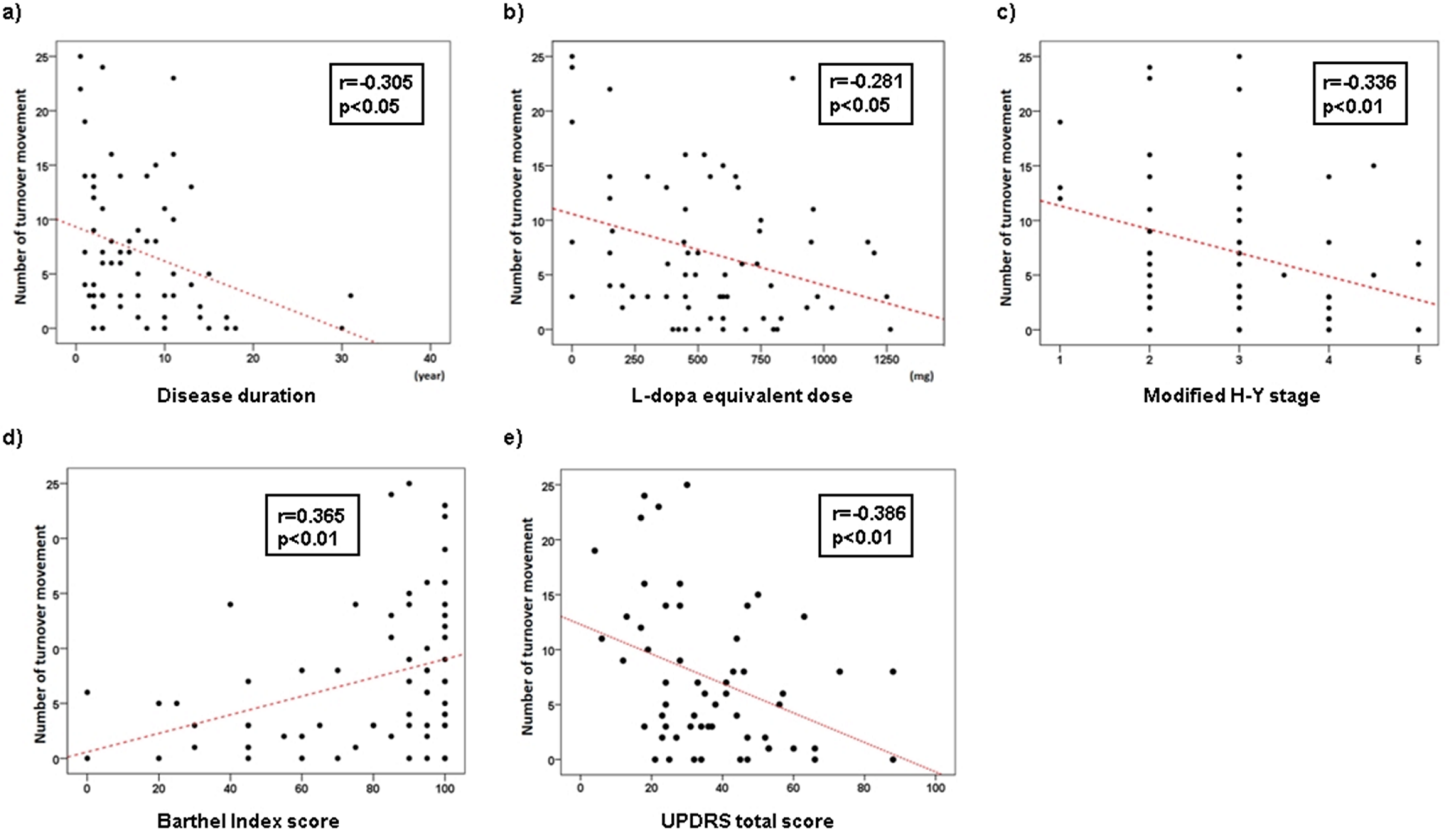
Correlations between number of turnover movements in bed and background factors. Number of turnover movements in bed correlated significantly with disease duration (a), L-dopa equivalent dose (b), modified H-Y stage (c), B-I (d), and total UPDRS score (e).

Disease duration, LED, mH-Y staging, B-I and UPDRS (total score, Part 2, and Part 3) correlated significantly with nocturnal parameters (Table 2). Number of turnover movements in bed correlated negatively with duration of disease (r = -0.305; p<0.05), LED (r = -0.281; p<0.05), mH-Y staging (r = -0.336; p<0.01), total score of UPDRS (r = -0.386; p<0.01), UPDRS Part 2 (r = -0.317; p<0.05), and UPDRS Part 3 (r = -0.310; p<0.05), and positively with B-I score (r = 0.365; p<0.01). Higher mH-Y stage and UPDRS score indicate poor function, whereas higher B-I score indicates better activities of daily living. The tendencies of correlation coefficients are thus similar. For total time recumbent, total time supine, and mean interval between turnover movements, tendencies of correlation coefficients were similar; i.e., patients with poor motor activity as indicated by mH-Y, B-I, and UPDRS scores tended to show longer time staying in bed, longer time staying supine, and longer interval between turnover movements. These relationships were similarly observed in disease duration and LED. Correlations between number of turnover movements and patient characteristics are shown in Fig 3.

**Table 2:** Correlation coefficients between nocturnal parameters and patient’s characteristics.

	Age	Disease duration	LED	mH-Y staging	Barthel index	UPDRS Total	UPDRS Part 1	UPDRS Part 2	UPDRS Part 3	UPDRS Part 4
Number of Turnovers	-.100	-.305^*^	-.281^*^	-.336^**^	.365^**^	-.386^**^	.019	-.317^*^	-.310^*^	-.217
Total Time for recumbent position	.039	.313^*^	.311^*^	.407^**^	-.425^**^	.286^*^	.152	.307^*^	.181	.111
Total Time for supine position	.230	.252^*^	-.071	.307^*^	-.435^**^	.317^*^	.098	.227	.255	-.005
Mean interval of turnovers	.121	.350^**^	.325^**^	.381^**^	-.410^**^	.412^**^	.000	.345^*^	.334^*^	.223

Values represent Spearman correlation coefficients.

*p<0.05

**p<0.01.

LED, L-dopa-equivalent dose; mH-Y scale, modified Hoehn-Yahr scale; UPDRS, Unified Parkinson’s Disease Rating Scale.

The frequency of turnover movements in bed for PD patients by quartile was as follows: 0–2 times, n = 17; 3–5 times, n = 16; 6–11 times, n = 16; and ≥12 times, n = 15. Predictive accuracies of mH-Y, B-I, and UPDRS for the 1st quartile (0–2 turnover movements) were obtained from receiver operated characteristics curve (ROC) analysis, with areas under the ROC curves of 0.685 (95% confidence interval (CI), 0.532–0.839), 0.698 (95%CI, 0.553–0.844), and 0.709 (95%CI, 0.559–0.858), respectively.

### Awareness of inability to turn in bed and actual number of turnover movements in bed

Relationships between subjective awareness of difficulty turning in bed as categorized by the PDSS-2 item 9 and nocturnal kinetic parameters are shown in Table 3. Although PD patients with a higher score for PDSS-2 item 9 tended to show longer total time recumbent and longer mean interval between turnover movements (p = 0.077 and p = 0.081, respectively), no significant difference was observed among the 5 groups categorized by PDSS-2 item 9 scores.

**Table 3:** Subjective awareness of difficulty turning in bed as evaluated by PDSS-2 item 9 and nocturnal kinetic parameters.

	Score 0 (Never)	Score1 (Occasionary)	Score2 (Sometimes)	Score3 (Often)	Score 4 (Very often)	P value
**Number of Turnovers, median (min-max)**	**5 (4–8)**	**5 (2–13)**	**6 (1–7)**	**6 (2–10)**	**3 (1–12)**	**0.077**
**Total Time for recumbent position, mean (SD) minutes**	**520.4(49.1)**	**514.4 (47.9)**	**540.2 (39.0)**	**489.0 (72.1)**	**563.2 (36.9)**	**0.771**
**Total Time for supine position, mean (SD) minutes**	**299.1(156.3)**	**346.0 (141.1)**	**322.6 (129.9)**	**337.3 (123.5)**	**277.0 (158.2)**	**0.133**
**Mean interval of turnovers, mean (SD) minutes**	**45.6 (22.8)**	**225.1 (204.4)**	**86.9 (85.6)**	**153.9 (173.9)**	**168.4 (205.7)**	**0.081**

**Fig 4:**
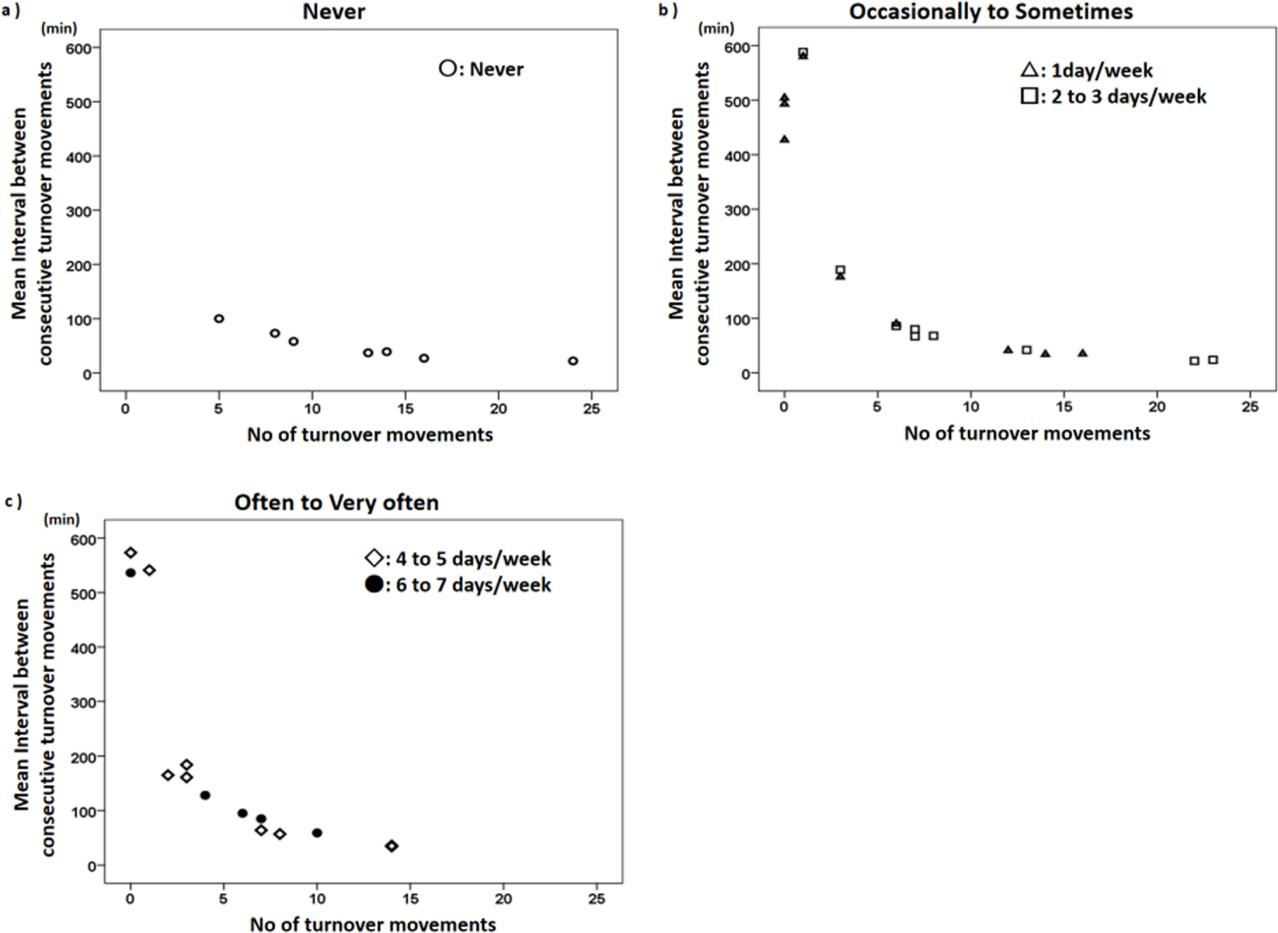
Subjective scoring of discomfort and immobility in bed as evaluated by PDSS-2 Item 9 and objective numbers of overnight turnover movements. Mean interval between consecutive turnover movements (minutes) as plotted against number of turnover movements in scattergrams by subjective scores as evaluated by PDSS-2 item 9: never aware of impairment of turnover movement (a); occasionally to sometimes (b); and often to very often (c). In patients with no subjective complaint, number of turnover movements overnight was ≥5 and mean interval between consecutive turnovers was <100 min (a). Among patients showing >5 turnover movements, various PDSS-2 Item 9 scores ranging from 0 to 4 (“never” to “very often”) were noted.

Scattergrams in Fig 4 show the relationship between number of turnover movements and mean interval between consecutive turnover movements. Visual analysis of data from patients who were never aware of impairment of turnover movement (PDSS-2 Item 9 Score = 0) showed ≥5 turnover movements overnight (Fig 4A). Patients who were often or very often aware of impaired turnover movement (PDSS-2 Item 9 Score = 3 or 4) showed a maximum of 15 turnover movements in overnight measurements. However, among patients showing 5–15 turnover movements, various PDSS-2 Item 9 scores ranging from 0 to 4 (“never” to “very often”) were noted. The number of turnover movements in bed was thus generally inconsistent with awareness of impaired turnover movement as evaluated by PDSS-2 Item 9 scores.

### Correlations between number of turnover movements in bed, sleepiness and depressive features

A total of 58 patients (90.6%) were receiving L-dopa and 6 patients (9.4%) were receiving other drugs. A total of 24 patients (37.5%) were receiving L-dopa alone, and 34 patients (53.1%) were receiving concomitant treatment with L-dopa and dopamine adjuvants. We constructed a model using age, LED, UPDRS, use of sleeping drugs, use of anti-psychotic drugs, use of dopamine agonists, and number of nocturnal turnover movements in bed, and investigated associations between each of these factors and outliers in ESS, PDSS-2, and BDI in multivariate logistic regression analysis (Table 4). Anti-psychotic drug use was significantly associated with ESS scores ≥10, and UPDRS was significantly associated with PDSS-2 scores ≥15. No factors were significantly associated with depressive features as evaluated by BDI score (>20). Number of nocturnal turnover movements in bed was not significantly correlated with BDI, ESS, or PDSS-2 abnormal score.

**Table 4:** Multivariate logistic regression analyses.

	ESS			PDSS-2			BDI		
	OR	(95%CI)	p	OR	(95%CI)	p	OR	(95%CI)	p
Age	0.951	(0.829–1.090)	0.471	0.785	(0.596–1.035)	0.087	1.004	(0.892–1.131)	0.941
LED	1.003	(0.999–1.007)	0.193	0.999	(0.995–1.003)	0.554	1.003	(0.999–1.007)	0.211
UPDRS	1.002	(0.943–1.064)	0.948	1.152	(1.027–1.291)	**0.016**	1.019	(0.967–1.075)	0.476
Sleeping drugs	0.103	(0.005–2.244)	0.148	1.293	(0.140–11.971)	0.821	3.156	(0.475–20.954)	0.232
Antipsychotics	46.183	(1.099–1941.5)	**0.045**	1.941	(0.091–41.363)	0.671	1.920	(0.209–17.648)	0.564
Dopamine agonist	2.442	(0.229–26.00)	0.459	0.599	(0.067–5.384)	0.647	0.833	(0.090–7.728)	0.872
Number of turnovers	1.174	(0.972–1.353)	0.105	1.062	(0.907–1.245)	0.454	0.924	(0.794–1.075)	0.331

ESS, Epworth Sleepiness Scale; PDSS-2, Parkinson’s Disease Sleep Scale-2; BDI, Beck Depression Inventory

For logistic regression analysis, ESS score ≥10, PDSS-2 score ≥15, and BDI score >20 were taken to indicate excessive daytime sleepiness, sleep disturbance, and moderate to severe depressive features, respectively.

## Discussion

Nocturnal parameters including number of turnover movements, time spent in a recumbent position in bed, time spent in a supine position in bed, and mean interval between consecutive turnover movements correlated significantly with disease duration, LED, mH-Y, UPDRS total score, and B-I (Table 2 and Fig 3). These results suggest that less frequent nocturnal turnover movements and longer period spent recumbent or supine are observed in PD patients showing deterioration in scores reflecting daytime immobility, greater disease duration, and LED. It is reasonable to assume that the degree of daytime immobility is also predictive of night-time motor disability. Our study demonstrated UPDRS total score as the most predictive for turnover movement impairment (area under the ROC curve, 0.709) and the number of turnover movements in bed correlated significantly with mH-Y, B-I, and UPDRS. However, the most significant correlation (UPDRS, -0.386) still yielded a low coefficient. Our study also demonstrated that number of turnover movements in bed was generally inconsistent with awareness of turnover movement impairment as evaluated by PDSS-2 Item 9 score (Fig 4), implying that nocturnal immobility should be assessed objectively with three-axis accelerometers rather than subjectively by self-report.

This study posed the question of whether night-time immobility affects quality of life among PD patients, but multivariate logistic regression analysis failed to demonstrate an association between number of turnover movement and outliers of ESS, PDSS-2, and BDI. Use of anti-psychotic drugs and UPDRS total score appeared to represent major determinants for outliers of ESS and PDSS-2, respectively. No factor, including number of turnover movements in bed, was significantly associated with BDI. We thus conclude that nocturnal immobility may not be a major determinant of daytime sleepiness, nor mood disorders including depressive features in PD patients. However, further studies are needed to answer this question. Bhidayasiri et al. recently demonstrated that nocturnal apomorphine infusion improved nocturnal hypokinesia in PD patients using a wearable three-axis accelerometer [11]. Rotigotine treatment reportedly improved UPDRS Part 2 sub-score and PDSS in a study by Trenkwalder et al. [19]. Prolonged-release ropinirole tablets reportedly improved objective quality of sleep in a study by Dusek et al. [23]. These drugs can be expected to ameliorate nocturnal symptoms. The real clinical significance of nocturnal turnover movement impairment needs to be investigated in further research focusing on how nocturnal turnover movement impairment can be ameliorated with adjustments in dopamine agonist medication and how such treatments can improve quality of life in PD patients. We therefore consider that the method of overnight monitoring with three-axis accelerometers reported in this study has great utility for such therapeutic intervention studies. In such studies, use of antipsychotic drugs and UPDRS score should be considered as important confounders.

A number of limitations need to be considered when interpreting the results of this study. First, we did not perform polysomnographic analysis. Our sensor-based monitoring of nocturnal movement can detect periods of wakefulness based on body position, but sleep stage in the recumbent position in bed cannot be evaluated. In future studies focusing on daytime sleepiness or mood disorders, polysomnography combined with a sensor-based kinetic method may provide comprehensive data. A second limitation concerns the definition of turnover movements in bed for this study, which was based on the method reported by Akamatsu et al. [8]. Sensors were set to pick up movements ≥30° around the posteroanterior body axis. Accordingly, any effects of movements involving corresponding rotations <30° during sleep would not have been identified in this study. The third limitation concerned the nature of this research as a cross-sectional, observational study.
